# Enzyme-Assisted Ultrasound Extraction of Bioactive Compounds from *Ziziphus jujuba* Mill. cv. ‘Junzao’ Fruits: Untargeted Metabolomic Profiling Reveals Enhanced Antioxidant Capacity

**DOI:** 10.3390/antiox15101244

**Published:** 2026-09-26

**Authors:** Yahui Yan, Chaoming Zhang, Dan Rui, Yongxia Tao, Zuoshan Feng

**Affiliations:** 1 College of Food Science and Pharmacy, Xinjiang Agricultural University, Urumqi 830052, China; 2 Postdoctoral Station of Horticulture, Xinjiang Agricultural University, Urumqi 830052, China

**Keywords:** untargeted metabolomics, *Ziziphus jujuba* Mill. cv. ‘Junzao’, ultrasound-assisted extraction, enzyme-assisted extraction, primary metabolites, secondary metabolites, antioxidant activity

## Abstract

Enzyme-assisted ultrasound extraction offers a synergistic dual-disruption strategy, yet its differential effects on the jujube metabolome and antioxidant capacity have not been systematically characterized. This study compared ultrasound-assisted extraction (UAE) alone (N), pectinase-assisted UAE (G), cellulase-assisted UAE (X), and composite enzyme-assisted UAE (GX) for metabolite recovery from *Ziziphus jujuba* Mill. cv. ‘Junzao’ fruits using untargeted metabolomics and multiple antioxidant assays. The GX group exhibited the highest total phenolic content (33.42 ± 0.73 mg GAE/g DW) and total flavonoid content (7.70 ± 0.18 mg RE/g DW), significantly exceeding those of the control (N). Untargeted metabolomic profiling annotated 839 metabolites classified into 16 chemical superclasses, with lipids and lipid-like molecules, amino acids and derivatives, and organic acids and derivatives being the most abundant categories. Multivariate analysis confirmed clear compositional separation between the enzyme-assisted groups and the control, with 107 differentially extracted metabolites (DEMs) identified across the three pairwise comparisons based on extraction efficiency differences. The GX group consistently showed the strongest antioxidant activity in 2,2′-azino-bis(3-ethylbenzothiazoline-6-sulfonic acid) (ABTS), 2,2-diphenyl-1-picrylhydrazyl (DPPH), ferric reducing antioxidant power (FRAP), and hydroxyl radical scavenging assays, and eight metabolites with significantly higher extraction yield showed significant positive correlations with multiple antioxidant indices in Spearman correlation analysis. These findings demonstrate that composite enzyme-assisted UAE achieved superior metabolite coverage and antioxidant potency through synergistic cell-wall deconstruction, providing a metabolomic framework for the green extraction of bioactive compounds from jujube fruits; however, further targeted validation is warranted.

## 1. Introduction

The growing global demand for natural antioxidants in functional foods, nutraceuticals, and pharmaceuticals has intensified the need for efficient recovery of bioactive compounds from plant matrices [1]. Phenolics, flavonoids, and other secondary metabolites derived from botanical sources exhibit superior biocompatibility and multi-target bioactivities compared with synthetic counterparts, making them central to current research in preventive nutrition and green chemistry [2,3]. However, the efficient, selective, and sustainable extraction of these compounds from complex plant tissues remains a core challenge in natural product chemistry and food processing. Conventional extraction techniques—including heat reflux extraction, solvent extraction, and maceration with organic solvents—have long served as industrial standards but are increasingly recognized as inadequate for modern requirements [4,5]. These methods typically require prolonged processing times (often >6 h), elevated temperatures (60–100 °C), and large volumes of organic solvents (methanol, ethanol, hexane), resulting in high energy consumption, substantial solvent waste, and a significant environmental burden [4,5]. More critically, harsh thermal conditions induce degradation, isomerization, or oxidation of thermolabile bioactive molecules, including anthocyanins and polyunsaturated fatty acid derivatives, thereby diminishing both yield and biological potency [6,7]. Moreover, the rigid cell-wall architecture of fruit tissues—composed of cellulose microfibrils embedded in a pectin-hemicellulose matrix—acts as a physical barrier that severely restricts solvent penetration and mass transfer, constituting the central methodological dilemma in current extraction science [8,9].

In recent years, several emerging technologies—ultrasound-assisted extraction (UAE), microwave-assisted extraction (MAE), supercritical fluid extraction (SFE), pressurized liquid extraction (PLE), and pulsed electric field (PEF)—have been developed and validated as promising alternatives [10,11]. Among these, UAE has attracted particularly extensive attention due to its operational simplicity, scalability, low equipment cost, broad solvent compatibility, and demonstrated effectiveness across diverse plant matrices [12]. Nevertheless, for fruit tissues rich in pectin and cellulose, UAE alone may be insufficient to achieve comprehensive cell-wall disintegration, leaving intracellular metabolites trapped within partially intact cellular compartments [13]. Extraction yields tend to plateau beyond a threshold sonication intensity, indicating that complementary strategies are required to overcome this structural barrier [14].

Enzyme-assisted extraction (EAE) represents a biocatalytic approach that targets the structural polymers of plant cell walls with high specificity under mild conditions (pH 4–6, 40–55 °C) and offers a biologically specific solution to this bottleneck [15,16]. Pectinase predominantly hydrolyzes the α-1,4-galacturonan backbone of pectin and associated structures in the middle lamella and primary cell wall; cellulase cleaves β-1,4-glycosidic bonds in cellulose microfibrils, fragmenting the structural scaffold; and their combination provides comprehensive wall deconstruction [17,18]. When enzymatic pretreatment is coupled with subsequent UAE, a synergistic dual-disruption mechanism emerges: enzymatic hydrolysis first creates microchannels in the middle lamella and primary wall, after which ultrasound further accelerates solvent permeation and mass transfer, generating a spatiotemporal synergy that promotes the deep-layer release of metabolites [19,20]. Critically, most existing literature has focused predominantly on process parameter optimization using conventional response metrics (TPC, TFC, or single-compound yield) but has rarely revealed specific metabolite classes preferentially released by different enzymatic strategies or the intrinsic relationships between specific metabolites and multidimensional antioxidant activity.

Untargeted metabolomics based on ultra-high-performance liquid chromatography coupled with high-resolution mass spectrometry (UHPLC-HRMS/MS) provides the analytical resolution necessary to shift this paradigm. By simultaneously annotating hundreds of metabolites without prior compound selection, this approach enables the construction of comprehensive extraction-dependent chemical fingerprints and the identification of differentially extracted metabolites (DEMs) through multivariate statistical analysis [21,22]. The few existing studies have applied metabolomics to compare conventional and ultrasound-assisted extraction methods [23,24], but systematic metabolomic evaluation of enzyme-assisted strategies—particularly the comparison of single versus composite enzyme approaches—has not been reported.

*Ziziphus jujuba* Mill. is one of the most economically important fruit species in China, with a cultivation history exceeding 4000 years [25]. Jujube fruits are a well-established medicine-food homologous resource rich in phenolic acids, flavonoid glycosides, triterpenoids, cyclic nucleotides, and polysaccharides, which collectively contribute to documented antioxidant, immunomodulatory, hepatoprotective, and anti-inflammatory activities [26,27]. Among the numerous cultivars, *Ziziphus jujuba* Mill. cv. ‘Junzao’ (Junzao) is widely cultivated in the Xinjiang region and is characterized by large fruit size, thick mesocarp tissue with abundant pectin and cellulose, and high phenolic content [28]. These structural and compositional features make Junzao an ideal model system for evaluating enzyme-assisted extraction pipelines.

However, the systematic effects of different enzyme-assisted extraction strategies on the metabolome and antioxidant capacity of Junzao remain elusive. We hypothesized that enzyme type and combination significantly shape both the metabolite fingerprint and the antioxidant potential of Junzao extracts, with composite enzyme-assisted UAE yielding the broadest metabolite coverage and strongest bioactivity. To address these gaps, the present study aimed to compare metabolite recovery of different extraction methods (N, G, X, and GX) in Junzao fruits; construct untargeted UHPLC-HRMS/MS metabolomic profiles and identify DEMs across extraction methods using multivariate analysis; and evaluate antioxidant capacity through multiple radical-scavenging assays while exploring structure–activity relationships among differential metabolites. These findings will provide new scientific perspectives and guidance for the green extraction of bioactive natural products.

## 2. Materials and Methods

### 2.1. Plant Materials and Chemicals

Fresh fruits were harvested at commercial maturity in October 2025 and purchased from Silk Road Xinwei Agricultural Products Cooperative located in Togergazi Township, Yutian County, Hotan, Xinjiang, China (36.84° N, 81.47° E). Voucher specimens were identified and deposited by the College of Food Science and Pharmacy, Xinjiang Agricultural University (Urumqi, China). The fruits were washed, pitted, oven-dried at 60 °C to constant weight in an electric hot-air circulating drying oven (Beijing Guangming Medical Instruments Co., Ltd., Beijing, China), pulverized using a high-speed universal crusher (FW-10, Beijing Yongguangming Medical Instrument Co., Ltd., Beijing, China), and sieved through a 100-mesh screen. The resulting powder was sealed and stored at 4 °C.

Pectinase (SPE-006, 10,000 U/mL) and cellulase (SPE-017, 10,000 U/g) were obtained from Cangzhou Xiasheng Enzyme Biotechnology Co., Ltd. (Cangzhou, China). Sodium nitrite, sodium hydroxide, and aluminum nitrate were purchased from Tianjin Xinbot Chemical Co., Ltd. (Tianjin, China). Methanol, anhydrous ethanol, and sodium carbonate were obtained from Tianjin Zhiyuan Chemical Reagents Co., Ltd. (Tianjin, China). Folin–Ciocalteu and hydroxyl radical scavenging assay kits (BC1320) were purchased from Solarbio Science & Technology Co., Ltd. (Beijing, China). All of the above reagents were of analytical grade. The ferric reducing antioxidant power (FRAP, A015-3-1) assay kit was obtained from Nanjing Jiancheng Bioengineering Institute (Nanjing, China). Rutin (≥98%), gallic acid (≥98%), ascorbic acid (Vc), 2,2-diphenyl-1-picrylhydrazyl (DPPH), and 2,2′-azino-bis(3-ethylbenzothiazoline-6-sulfonic acid) (ABTS) were purchased from Shanghai Yuanye Biotechnology Co., Ltd. (Shanghai, China). HPLC-grade formic acid, methanol, and acetonitrile were obtained from Shanghai Aladdin Biochemical Technology Co., Ltd. (Shanghai, China), Merck (Darmstadt, Germany), and Shanghai Xingkegao Alcohol Solvents Co., Ltd. (Shanghai, China), respectively.

### 2.2. Extraction Procedures

#### 2.2.1. Ultrasound-Assisted Extraction (N)

UAE employed a slightly modified method previously described by Chianese et al. [29]. Jujube powder (2.0 g) was accurately weighed using an electronic balance (ME204/02, Mettler-Toledo Instruments Co., Ltd., Shanghai, China) and suspended in 20 mL of 80% (*v*/*v*) ethanol−water solution. The mixture was subjected to ultrasound-assisted extraction for 45 min using an ultrasonic cleaner (KQ-400DE, Kunshan Ultrasonic Instruments Co., Ltd., Kunshan, China) at room temperature. After extraction, the mixture was centrifuged (4K15, Sigma Laboratory Centrifuges, Inc., Osterode am Harz, Germany) at 12,000 rpm for 10 min. The extraction was repeated twice under the same conditions, and the supernatants were combined and brought to a final volume of 50 mL with 80% ethanol–water solution to prepare a test solution with a concentration of 40 mg/mL. An aliquot of the supernatant was filtered through a 0.22 μm microporous membrane filter for subsequent assays. The dry extract yield was determined by the weighing method: 50 mL of each extract was concentrated using a rotary evaporator (N-1001, Shanghai Ailang Instruments Co., Ltd., Shanghai, China) and freeze-dried in a vacuum freeze-dryer (SCIENTZ-10N/C, Ningbo Xinzhi Biotechnology Co., Ltd., Ningbo, China) until constant weight was reached; the yield was expressed in *g*/*g* × 100% of dry powder.

#### 2.2.2. Pectinase-Assisted UAE (G)

EAE-UAE employed a slightly modified method previously described by Lu et al. [30]. Jujube powder (2.0 g) was soaked in 10 mL of a citric acid aqueous solution (pH 5.5), and 20 mg of pectinase was added. The mixture was incubated at 55 °C for 2 h in an electrically heated constant-temperature water bath (DZKW-S-4, Beijing Yongguangming Medical Instrument Co., Ltd., Beijing, China), with constant stirring at 500 rpm using a magnetic stirrer (VELP Scientifica S.r.l., Usmate Velate, Italy). The enzyme was subsequently inactivated by heating at 90 °C for 10 min, and the mixture was cooled to room temperature, then dried it. The hydrolyzed material was then extracted using the ultrasound procedure described in Section 2.2.1.

#### 2.2.3. Cellulase-Assisted UAE (X)

The procedure was identical to that in Section 2.2.2, except that cellulase (20 mg) was used instead of pectinase. All other enzymatic hydrolysis conditions and ultrasound extraction parameters were the same as those described in Section 2.2.2.

#### 2.2.4. Composite Enzyme-Assisted UAE (GX)

The procedure was identical to that in Section 2.2.2, except that a composite enzyme preparation consisting of pectinase and cellulase at a 1:1 (*w*/*w*) ratio was used at a total dosage of 20 mg. All other enzymatic hydrolysis and ultrasound extraction parameters were the same as those described in Section 2.2.2.

### 2.3. Determination of Total Phenolic Content (TPC)

TPC was determined using the Folin–Ciocalteu colorimetric method with minor modifications [31]. Briefly, 1.0 mL of appropriately diluted extract was mixed with 0.5 mL of Folin–Ciocalteu reagent and allowed to react for 8 min. Then, 1.5 mL of 20% (*w*/*v*) sodium carbonate solution was added. The mixture was incubated at room temperature for 24 h in the dark. Absorbance was measured at 765 nm using a UV-Vis spectrophotometer (UV-2550, Shimadzu Instruments Co., Ltd., Kyoto, Japan). A calibration curve was constructed using gallic acid over the concentration range of 3.125–50 μg/mL (Figure S1A). Results were expressed as milligrams of gallic acid equivalents per gram of dry weight (mg GAE/g DW).

### 2.4. Determination of Total Flavonoid Content (TFC)

TFC was determined using the aluminum nitrate-sodium nitrite colorimetric method with slight modifications [31]. Briefly, 1.0 mL of extract was mixed with 0.3 mL of 5% (*w*/*v*) NaNO_2_ solution and allowed to stand for 6 min. Subsequently, 0.3 mL of 10% (*w*/*v*) Al(NO_3_)_3_ solution was added, and the mixture was allowed to stand for another 6 min. Then, 4.0 mL of 5% (*w*/*v*) NaOH solution and 4.4 mL of 80% (*v*/*v*) methanol were added. The mixture was allowed to stand for 15 min, and the absorbance was measured at 510 nm. A calibration curve was constructed using rutin over the concentration range of 0.05–1.0 mg/mL (Figure S1B). Results were expressed as milligrams of rutin equivalents per gram of dry weight (mg RE/g DW).

### 2.5. Untargeted Metabolomic Analysis by UHPLC-HRMS/MS

#### 2.5.1. Sample Preparation

Extracts stored at −80 °C were thawed at room temperature until no ice crystals remained and vortexed for 10 s to ensure homogeneity. An aliquot of 200 μL of each sample was transferred into a 1.5 mL microcentrifuge tube, followed by the addition of 200 μL of 70% methanol (*v*/*v*) containing 0.25 mg/L lidocaine as an internal standard [32]. The mixture was vortexed for 5 min and then centrifuged at 12,000 rpm for 3 min at 4 °C. The supernatant was filtered through a 0.22 μm PTFE membrane filter and stored in injection vials for UHPLC-HRMS/MS analysis. Quality control (QC) samples were prepared by pooling equal aliquots of all experimental samples and were analyzed at regular intervals throughout the analytical sequence to monitor system stability and data quality.

#### 2.5.2. Chromatographic and Mass Spectrometric Conditions

Chromatographic separation was performed on a Vanquish UHPLC system (Thermo Fisher Scientific, Waltham, MA, USA) equipped with a Waters ACQUITY UPLC HSS T3 column (1.8 μm, 2.1 mm× 100 mm; Waters Corporation, Milford, MA, USA) maintained at 40 °C. The mobile phase consisted of 0.1% (*v*/*v*) formic acid in water (A) and 0.1% (*v*/*v*) formic acid in acetonitrile (B). The gradient elution program was as follows: 0–2.0 min, 5–25% B; 2.0–4.0 min, 25–99% B; 4.0–4.5 min, 99% B; 4.5–4.6 min, 99–5% B; 4.6–6.0 min, 5% B. The flow rate was 0.40 mL/min, and the injection volume was 4 μL.

Mass spectrometric detection was performed on a Q Exactive HF-X high-resolution mass spectrometer (Thermo Fisher Scientific, Waltham, MA, USA) equipped with a heated electrospray ionization (HESI) source operating in both positive and negative ion modes. The key parameters were set as follows: spray voltage, +3500 V (positive mode) and −3200 V (negative mode); sheath gas flow rate, 30 arbitrary units (Arb); auxiliary gas flow rate, 5 Arb; ion transfer tube temperature, 320 °C; and vaporizer temperature, 300 °C. Full-scan MS data were acquired in the range of *m*/*z* 84–1250 with a resolution of 35,000 FWHM. Data-dependent MS/MS spectra were acquired at a resolution of 17,500 with normalized collision energies (NCE) of 30, 40, and 50 eV.

#### 2.5.3. Metabolomics Data Processing and Multivariate Statistical Analysis

The total ion chromatograms (TICs) of the QC samples demonstrated excellent reproducibility and analytical stability (Figure S2). As shown in Figure S3, representative TICs of the four extraction groups were acquired in both negative and positive ion modes. Raw data files were converted to mzML format using ProteoWizard MSConvert (version 3.0). Peak detection, alignment, and retention time correction were performed using the XCMS package (version 4.0) in R. Metabolites annotation was performed by matching accurate mass, retention time, and MS/MS fragmentation patterns against an in-house database (Metware Database, MWDB), and no metabolite was isolated or structurally confirmed in this study. Mass tolerances were set at ≤25 ppm for both precursor ions and fragment ions. Only metabolites with a coefficient of variation (CV) <50% in QC samples and composite identification scores ≥0.5 were retained. Metabolite identification confidence levels were assigned to Level 1a (high-confidence matching) and Level 1b (moderate-confidence matching), based on the MS1, retention time, and MS/MS fragment matches results obtained from comparative analysis with reference standards under identical conditions in the MWDB database. The identifiers given were the in-house database accession codes of the annotation pipeline, and annotated metabolites were assigned to chemical superclasses and classes using the ClassyFire hierarchical taxonomy based on their annotated structures. Quantitative results in untargeted metabolomics were relative, based on integrated MS1 peak areas after internal-standard (lidocaine) normalization and QC-based batch correction. Peak areas are proportional to the amount of each metabolite recovered in each extract, but do not represent absolute concentrations.

Hierarchical clustering analysis (HCA) was performed using Euclidean distance and average linkage methods with Z-score normalized data to visualize metabolite accumulation patterns. Principal component analysis (PCA) was conducted to assess overall metabolic variations among the different extraction groups. Orthogonal partial least squares-discriminant analysis (OPLS-DA) was performed to identify discriminating metabolites; DEMs were defined as those with VIP ≥ 1, |log2 fold change| ≥ 1, and *p* < 0.05 in univariate statistical analysis [33]. In the context of this extraction study, DEMs refer to compounds that show significantly different recovery efficiencies among extraction methods, reflecting differences in cell wall disruption and mass transfer rather than differences in biosynthesis. Fold-change calculations represent extraction efficiency differences, not biological regulation. K-means clustering, heatmaps, Venn diagrams, and Spearman correlation analysis of DEMs were conducted using the Metware Cloud Platform (Metware Biotechnology Co., Ltd., Wuhan, China) and R software (version 4.3.0).

### 2.6. In Vitro Antioxidant Activity Assays

#### 2.6.1. DPPH and ABTS Radical Scavenging Assay

The DPPH and ABTS radical scavenging activities were determined according to a previously described method, with slight modifications [34]. The extracts were diluted to 200 μg/mL. Briefly, 0.5 mL of extract was mixed with 2.0 mL of 0.1 mM DPPH solution in anhydrous ethanol. The mixture was incubated at room temperature for 30 min in the dark. Absorbance was measured at 517 nm. The ABTS working solution was prepared by mixing 7.0 mM ABTS stock solution with 2.45 mM potassium persulfate (1:1, *v*/*v*) and incubating the mixture in the dark at room temperature for 12–16 h. The solution was then diluted with anhydrous ethanol to obtain an absorbance of 0.70 ± 0.02 at 734 nm. Then, 0.2 mL of extract was added to 3.0 mL of ABTS working solution, and the mixture was incubated at room temperature for 6 min in the dark. Absorbance was measured at 734 nm. A concentration of 10 μg/mL Vc was used as the positive control for the ABTS and DPPH tests. The *inhibition rate* (%) was calculated as follows:

(1) $$Inhibition\;rate\;(\%)=(1-\frac{A_{1}-A_{2}}{A_{0}})\times 100$$

where *A*_0_ is the absorbance of the mixed DPPH/ABTS solution and the corresponding solvent; *A*_1_ is the absorbance of the mixed sample solution and DPPH/ABTS solution; and *A*_2_ is the absorbance of the mixed sample solution and the corresponding solvent.

#### 2.6.2. FRAP Assay

The FRAP assay was performed using a commercial assay kit (No. A015-3-1) following the manufacturer’s instructions [35]. The sample extract was diluted to 5 mg/mL. Briefly, 5 μL of extract was mixed with 180 μL of FRAP working reagent and incubated at 37 °C for 5 min. Absorbance was measured at 593 nm. A calibration curve was constructed using FeSO_4_·7H_2_O over the concentration range of 0–1.5 mM (y = 0.407x + 0.0055, R^2^ = 0.9996). Results were expressed as micromoles of Fe^2+^ equivalents per gram of dry weight. A 25 μg/mL Vc solution was used as the positive control. The *T-AOC* (μmol/g) was calculated according to the kit instructions:

(2) $$T-AOC(mol/g)=\frac{\Delta A\times V}{W}\times 1000$$

where *ΔA* is the concentration of the sample extract obtained from the calibration curve (mM); *V* is the total volume of the sample extract (L); and *W* is the mass of the sample (g).

#### 2.6.3. Hydroxyl Radical Scavenging Assay

The hydroxyl radical scavenging capacity was measured using a commercial assay kit (No. BC1320) following the manufacturer’s protocol [36]. Blank, control, and sample reaction tubes were prepared sequentially according to the manufacturer’s instructions. After vortex mixing, all tubes were incubated at 37 °C for 60 min and then centrifuged at 12,000 rpm at room temperature for 10 min. The absorbance of the supernatants was measured at 536 nm. A 25 μg/mL Vc solution was used as the positive control. The *scavenging rate* (%) was calculated according to the kit instructions:

(3) $$Scavenging\;rate(\%)=\frac{A_{S}-A_{C}}{A_{b}-A_{C}}\times 100$$

where *A_s_* is the absorbance of the sample tube, *A_c_* is the absorbance of the control tube, and *A_b_* is the absorbance of the blank tube.

### 2.7. Statistical Analysis

All measurements were performed in triplicate using three biological replicates, and the results were expressed as mean ± standard deviation (SD). One-way analysis of variance (ANOVA), followed by Duncan’s multiple range test, was performed to determine significant differences among extraction methods at *p* < 0.05. Statistical analyses and figures were prepared using GraphPad Prism 9.0 (GraphPad Software, San Diego, CA, USA) and R software (version 4.3.0).

## 3. Results

### 3.1. The Effect of Different Extraction Methods on TPC and TFC in Extracts

The TPC values ranged from 24.27 ± 0.85 to 33.42 ± 0.73 mg GAE/g DW, whereas the TFC values ranged from 5.92 ± 0.17 to 7.70 ± 0.18 mg RE/g DW across the N, G, X, and GX extracts (Figure 1A,B). All enzyme-assisted extraction groups exhibited higher TPC and TFC values than the control group (N), with the highest levels observed in the GX group. These results suggest that enzyme-assisted pretreatment facilitates the release of phenolics and flavonoids, and that composite enzyme-assisted extraction is the most effective approach for enriching bioactive compounds.

### 3.2. Overview of Metabolomic Profiles Across Four Extraction Methods

To comprehensively characterize the metabolomic differences among Junzao fruit extracts prepared by four extraction methods, untargeted metabolomic profiling was performed using UHPLC-HRMS/MS on the N, G, X, and GX groups, each with three biological replicates. A total of 839 metabolites were identified and classified into 16 chemical superclasses (Figure 2A). Of the 839 annotated metabolites, 555 were assigned to Level 1a and 284 to Level 1b; the complete identification evidence is presented in Table S1. Among these, lipids and lipid-like molecules were the most abundant category (18.12%), followed by amino acids and derivatives (17.76%), organic acids and derivatives (16.33%), and alkaloids and derivatives (13.23%). Other major categories included benzenoids (7.39%), nucleotides and derivatives (5.84%), flavonoids (4.05%), terpenoids (3.58%), and lignans and coumarins (1.79%), while the remaining metabolites were distributed across other classes. These results reflect the broad chemical diversity of jujube fruit metabolites.

The HCA heatmap revealed distinct release patterns of metabolites among the four extraction groups, with biological replicates within each group clustering closely together, indicating good analytical reproducibility (Figure 2B). PCA was performed to visualize the overall metabolomic variation among the four extraction methods. The first two principal components accounted for 31.86% (PC1) and 12.06% (PC2) of the total variance, respectively (Figure 2C). The PCA score plot showed clear separation between the enzyme-assisted extraction groups (G, X, and GX) and the control group (N), indicating that enzymatic pretreatment substantially altered the metabolite profile of the jujube extracts.

K-means clustering analysis was performed to characterize metabolite release patterns among the four extraction groups, and all metabolites were classified into nine distinct clusters (Class 1–9), each representing a unique release pattern (Figure 2D, Table S2). Several clusters showed markedly higher relative abundance in one or more enzyme-assisted groups than in the control group, whereas others showed the opposite trend or relatively stable levels. These results highlight the diverse and complex effects of different enzymatic treatments on the jujube metabolome. Among the previously reported marker constituents of *Ziziphus jujuba*, (+)-catechin, quercetin, kaempferol, caffeate, quinic acid, ferulic acid, oleanolic acid, betulinic acid, pomolic acid, and cAMP and cGMP were annotated in the present dataset (Table S1). Of these, cAMP consistently exhibited higher extraction yields in the enzyme-treated groups (G, X, and GX) than in the control (N). Caffeate and ferulic acid showed higher extraction in GX than in N, and quercetin showed higher extraction in X than in N. The remaining compounds showed no significant differences, indicating that enzymatic cell-wall disruption is compound-selective rather than uniformly enhancing recovery.

### 3.3. OPLS-DA Analysis of Compositional Differences in Extracts from Different Extraction Methods

To identify metabolites showing significantly different extraction efficiencies among enzyme-assisted and control methods, OPLS-DA was performed for three pairwise comparisons: G vs. N, X vs. N, and GX vs. N. All models showed good fit and predictive ability, and permutation tests with 200 iterations confirmed their robustness: G vs. N (R^2^X = 0.592, R^2^Y = 1, Q^2^ = 0.929), X vs. N (R^2^X = 0.443, R^2^Y = 1, Q^2^ = 0.735), and GX vs. N (R^2^X = 0.509, R^2^Y = 1, Q^2^ = 0.871) (Figure S4). The high R^2^Y and Q^2^ values, together with successful permutation validation, indicated that the OPLS-DA models were robust and not overfitted. The OPLS-DA score plots showed clear separation between each enzyme-assisted group and the control group (N), confirming the discriminative power of the models (Figure 3A).

Volcano plots illustrated the distribution of higher extraction yield and lower extraction yield DEMs in each comparison (Figure 3B). Among them, 23 DEMs, including 12 metabolites with significantly higher extraction yield and 11 with significantly lower extraction yield, were identified in the GX vs. N comparison, and 20 DEMs, including 10 with significantly higher extraction yield and 10 with significantly lower extraction yield, were identified in the X vs. N comparison. Among the three comparisons, the G vs. N comparison yielded the highest number of DEMs (80, including 18 metabolites showing enhanced extraction and 62 showing reduced extraction). In total, 107 DEMs were identified across the three comparisons. These DEMs were mainly assigned to amino acids and derivatives (29), organic acids and derivatives (13), alkaloids and derivatives (11), benzenoids (10), lipids and lipid-like molecules (10), nucleotides and derivatives (9), flavonoids (9), and others (16) (Table S3).

A Venn diagram analysis was employed to evaluate the overlap of DEMs among the three pairwise comparisons (Figure 3C). Only one DEM, MEDP2131, was efficiently extracted by all three enzymatic methods compared to the control (G vs. N, X vs. N, and GX vs. N). In addition, 70, 11, and 15 DEMs were uniquely responsive to the G, X, and GX treatments, respectively, reflecting the specificity of the different enzymatic cell-wall disruption strategies.

To highlight metabolites that showed consistent enhancement in extraction efficiency across all enzyme-assisted methods, 20 metabolites were selected based on the criterion that their relative abundances in all three enzyme-assisted groups (G, X, and GX) were higher than those in the control group (N). These metabolites represent compounds that are effectively released by enzymatic pretreatment regardless of enzyme type, suggesting they were broadly entrapped within cell wall structures that are commonly disrupted by both pectinase and cellulase. Box plots presented the raw signal intensities of these 20 DEMs across the four groups (Figure S5). The chemical classification and relative abundance of these 20 representative metabolites were visualized using a hierarchical clustering heatmap, including four organic acids, two nucleotides and derivatives, two phenolic acids, two flavonoids, two amino acids and derivatives, two free fatty acids, and six others (Figure 3D). These results indicate that enzymatic pretreatment selectively enhanced the release or accumulation of specific classes of metabolites in Junzao fruit, providing new insights into optimization of the extraction process and targeted enrichment of functional components in this cultivar.

### 3.4. Antioxidant Activity of Different Extracts and Their Correlation with Key Metabolites

The dry extraction yields for the N, G, X, and GX groups were 38.95%, 57.55%, 56.57%, and 60.16%, respectively. The antioxidant capacities of Junzao fruit extracts obtained by the four extraction methods were evaluated using ABTS, DPPH, FRAP, and hydroxyl radical scavenging assays (Figure 4A–D). For ABTS radical scavenging activity (Figure 4A), G and GX enzyme-assisted extraction groups exhibited significantly enhanced antioxidant activities compared with the control group (N) (** *p* < 0.01; *** *p* < 0.0001). Among them, the GX group showed the highest scavenging rate (49.73%), followed by G (46.22%) and X (42.52%), all significantly higher than that of the control group (N, 39.88%). A similar trend was observed for DPPH radical scavenging (Figure 4B), in which the GX group exhibited markedly greater scavenging capacity, whereas the X and G groups showed no significant difference compared with N.

In the FRAP assay (Figure 4C), the GX group exhibited no difference from the Vc group but a significant difference from the N group, whereas the G and X groups did not differ significantly from the N group, indicating that the composite enzyme-assisted extract contained higher levels of electron-donating antioxidants. Similarly, the results for hydroxyl radical scavenging activity showed that all enzyme-assisted groups performed better than the control group(Figure 4D). Among them, the GX group again showed the highest activity (54.30%), with a scavenging rate slightly higher than that of the Vc group, followed by X (40.76%), G (33.68%), and N (28.94%). Overall, the GX extraction method consistently yielded extracts with the strongest antioxidant activities across all four assays, suggesting that composite enzyme treatment was more effective than single-enzyme treatments in disrupting the jujube fruit matrix and that the combined pectinase and cellulase treatment exerted a synergistic effect in releasing antioxidant compounds.

To elucidate the potential relationships between metabolite extraction yield and antioxidant capacity, Spearman correlation analysis was performed between the 20 higher yield DEMs and six biochemical parameters, including TPC, TFC, ABTS, DPPH, FRAP, and hydroxyl radical scavenging activity (Figure 4E, Table S5). The resulting correlation heatmap revealed that eight metabolites were positively correlated with all four antioxidant activities (*p* < 0.05), including MW0009620 (1,2,3-trihydroxybenzene), MW0007535 (maclurin), WAYP000599 (N-(1-deoxy-1-fructosyl)histidine), MEDN0031 (pyroglutamic acid), MWSMCE220 (D-glucono-1,5-lactone), MW0000440 (seneciphylline), MEDP2131 (L-isoserine), and MEDL02717 (traumatin). These correlation results provide preliminary evidence linking the metabolite-level changes induced by enzyme-assisted extraction to the functional antioxidant properties of the resulting extracts and highlight candidate bioactive compounds for future targeted validation studies.

## 4. Discussion

Plant extraction research has long relied on TPC, TFC, and chemical antioxidant assays (DPPH and ABTS) to evaluate extraction efficiency [37]. However, TPC and TFC are group-level colorimetric indicators that cannot resolve the actual compound composition of an extract. The Folin–Ciocalteu assay is susceptible to interference from reducing sugars, ascorbic acid, amino acids, browning products, and other reductive species; therefore, the measured TPC does not necessarily reflect the true phenolic content [38]. Similarly, the aluminum nitrate-sodium nitrite colorimetric method responds variably to flavonoids depending on hydroxyl position, glycosylation degree, and backbone structure, and it does not uniformly capture all flavonoid subclasses [39]. Cases in which TPC or TFC increases without a proportional improvement in antioxidant activity are well documented, because bioactivity depends on the identities, proportions, and interactions of specific compounds rather than on aggregate quantities alone [40,41]. Moreover, exclusive reliance on TPC/TFC overlooks non-phenolic contributors to antioxidant activity, such as ascorbic acid, glutathione, nitrogen-containing compounds, glycosylated products, and certain organic acids. Thus, single-metric optimization reduces extraction to increasing one numerical value without clarifying which compounds are released, which are degraded, and which actually drive bioactivity.

Untargeted metabolomics based on UHPLC-HRMS/MS can annotate hundreds to thousands of metabolites in a single run, providing a chemical fingerprint-based evaluation and shifting extraction assessment from bulk quantification toward metabolite profiling and functional correlation analysis [42]. In the present study, 839 metabolites were annotated and 107 DEMs were identified. Distinct metabolic fingerprints separated the four extraction groups, demonstrating that enzymatic pretreatment altered not only individual components but the overall chemical space of Junzao extracts. More importantly, the metabolomics data showed that most DEMs were not polyphenols or flavonoids, as conventional wisdom might suggest, but rather amino acids, lipids, organic acids, and alkaloids. This finding suggests that cell wall disruption releases a much broader chemical repertoire than commonly assumed and that enhanced antioxidant activity may result from multi-class synergistic effects rather than from polyphenol enrichment alone.

From a mechanistic perspective, this composition pattern is physiologically plausible. Amino acids, particularly those with sulfur-containing (cysteine and methionine), aromatic (tryptophan, tyrosine, and phenylalanine), or imidazole (histidine) side chains, possess intrinsic radical scavenging, metal-chelating, and reducing capacities [43,44]. Lipid-class DEMs may include membrane-derived phospholipids and fatty acid derivatives with peroxyl radical-quenching or lipoxygenase-inhibitory properties [45], whereas organic acids such as citric, malic, and oxalic acids may modulate extract pH and thereby indirectly enhance antioxidant capacity [46]. Alkaloids may also contribute through radical-scavenging activity associated with nitrogen lone pairs, aromatic ring systems, and hydroxyl substituents [47]. Compared with published jujube metabolomics studies, the present work extends the application of metabolomics to extraction-process evaluation in jujube fruits, representing a methodological advance for extraction method selection in this species. Future validation should include (1) targeted quantification of key DEMs using authenticated standards and (2) activity-guided fractionation to verify the bioactivity contributions of specific components. 

Enzyme-assisted ultrasound extraction can be viewed as the coupling of physical and biocatalytic forces and is especially advantageous for fruits with a thick mesocarp and high pectin content [48,49]. Junzao fruit has a pronounced cell wall architecture and an abundant pectin matrix; accordingly, a portion of phenolic acids, flavonoids, amino acids, organic acids, and other small molecules is likely present in cell-wall-adsorbed, embedded, or physically entrapped forms [50]. Such synergistic effects have been documented in multiple systems, including grape pomace [51], peach pomace [52], pomegranate peels [53], and various medicinal plants [54,55]. In the present study, GX consistently consistently exhibited superior antioxidant activity compared with single-enzyme treatments. Specifically, DPPH and ABTS radical scavenging activities followed the order GX > G > X > N, hydroxyl radical scavenging capacity followed the order GX > X > G > N, and FRAP-based antioxidant activity followed the order GX > G > N > X. This supported the complementarity of pectinase and cellulase in dismantling distinct cell-wall components, thereby achieving broader wall deconstruction while minimizing localized overprocessing.

Among the metabolites identified by Spearman correlation analysis as significantly associated with antioxidant capacity, some have been confirmed in the literature to possess strong antioxidant activity due to their structural characteristics. 1,2,3-trihydroxybenzene (MW0009620) contains adjacent polyhydroxyl groups that can scavenge free radicals through both hydrogen atom transfer and single-electron transfer mechanisms [56]. Maclurin (MW0007535) contains a polyhydroxylated A-ring system and a conjugated carbonyl group, which together form an effective radical-scavenging scaffold [57]. N-(1-Deoxy-1-fructosyl)histidine (WAYP000599), an Amadori rearrangement product, may be formed through a Maillard-type reaction between a reducing sugar and a free amino acid under localized high-temperature conditions induced by ultrasonic cavitation; it may exhibit moderate reducing power and metal-chelating activity [58]. Pyroglutamic acid (MEDN0031), with its gamma-lactam ring structure, is chemically stable, and its free carboxyl group may chelate transition metal ions (Fe^2+^, Cu^2+^), thereby contributing to indirect antioxidant activity in metal-catalyzed lipid peroxidation systems [59]. D-glucono-1,5-lactone (MWSMCE220) has been reported to function as a metal-chelating antioxidant by binding iron and copper, thereby preventing the metal-catalyzed production of hydroxyl radicals from superoxide and hydrogen peroxide [60]. Traumatin (MEDL02717), a fatty acid derivative, has been reported to induce traumatic acid accumulation upon plant tissue damage, thereby attenuating membrane phospholipid peroxidation and conferring protection against reactive oxygen species (ROS) generation [61]. Previous chemical studies on Junzao have disproportionately focused on rutin, isoquercitrin, and other flavonoid glycosides [62], while overlooking the contributions of simple phenolics, non-phenolic antioxidants, and reaction intermediates.

The integration of composite enzyme-assisted extraction, untargeted UHPLC-HRMS/MS, and in vitro antioxidant assays in this study provides a higher-resolution framework for process comparison than conventional TPC/TFC-based evaluation. The GX extraction system shows potential for producing functional raw materials from high-pectin fruits; however, industrial implementation will require systematic optimization of key process parameters, using both chemical yield and biological activity as dual endpoints. The metabolomics data generated here may guide the preparation of standardized Junzao extracts with defined composition and traceable bioactivity for use in dietary supplements, functional foods, or cosmetic ingredients. Nevertheless, translation from in vitro antioxidant activity to in vivo efficacy will require assessment of bioavailability, validation in cellular models, and confirmation of digestive stability and intestinal metabolism [63].

## 5. Conclusions

This study systematically compared single-enzyme pretreatments (G and X) and composite enzyme pretreatment (GX), all combined with ultrasound-assisted extraction, were systematically compared in terms of metabolite recovery from Junzao fruits. The results showed TPC and TFC were highest in the GX extract and were significantly higher in all enzyme-assisted groups than in the N group. Untargeted UHPLC-HRMS/MS profiling annotated 839 metabolites, and identified 107 DEMs, which were predominantly amino acids, lipids, organic acids, and alkaloids. In the four in vitro assays performed, the GX extract showed the highest activity; however, the differences among the enzyme-assisted groups were varied across assays, with X and G not differing significantly from N in the DPPH and FRAP assays. Eight annotated metabolites showed significant positive Spearman correlations with multiple antioxidant indices; these associations are hypothesis-generating and require confirmation with authentic standards. Overall, this study demonstrates that composite enzyme-assisted ultrasound extraction is a superior green strategy for recovering bioactive compounds from jujube fruits and provides a robust metabolomic framework linking enzymatic cell-wall deconstruction to enhanced antioxidant potency, thereby offering practical guidance for optimizing functional ingredient extraction from *Ziziphus jujuba* fruits.

Several limitations of this study should be acknowledged. First, metabolite identities are putative: annotation relied on accurate mass, retention time, and MS/MS database matching, and no metabolite was isolated or structurally confirmed by NMR. Second, quantification was based on relative peak areas rather than absolute concentrations, so the reported fold changes indicate differences in extraction efficiency and not absolute concentrations. Third, the antioxidant evaluation was limited to in vitro chemical assays; no cellular or in vivo experiments were performed. Fourth, all results were obtained from a single cultivar (‘Junzao’) harvested from a single geographical origin, which may limit the generalizability of the findings.

## Figures and Tables

**Figure 1:**
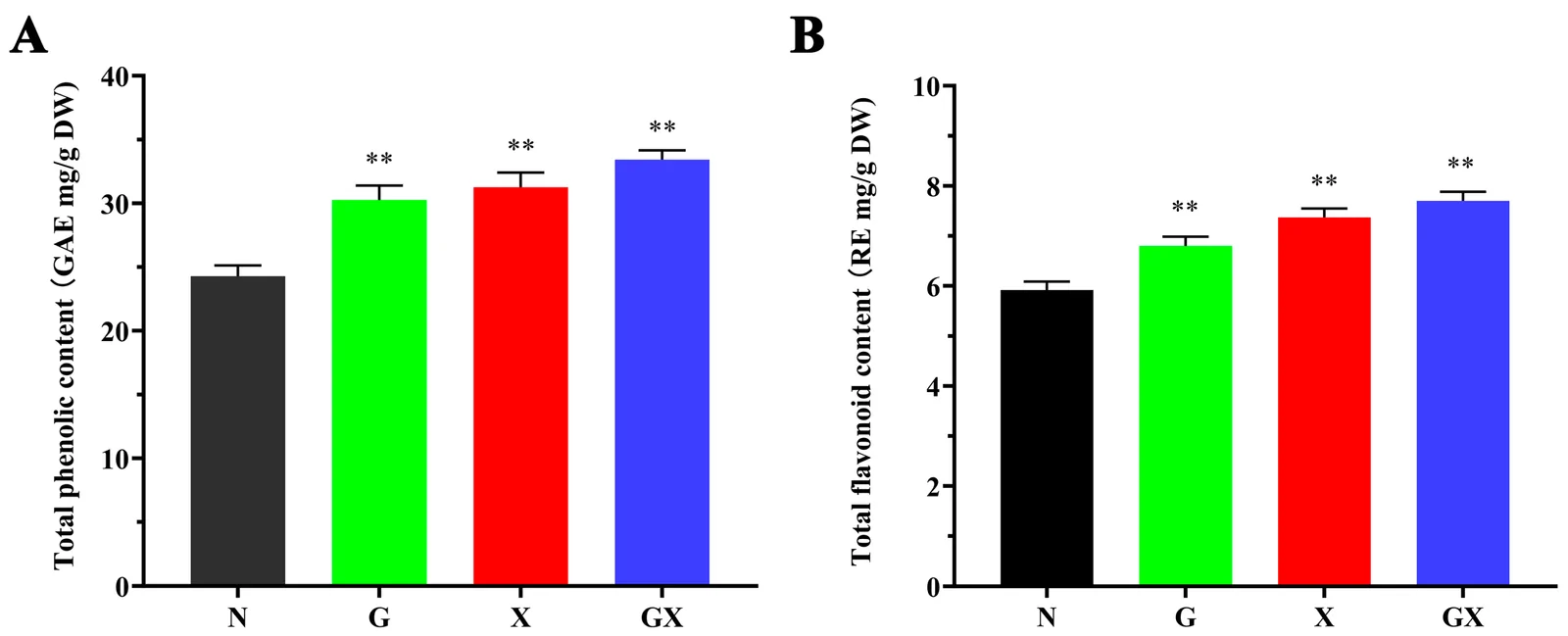
Comparison of total phenolic (TPC) and total flavonoid (TFC) contents in *Ziziphus jujuba* Mill. cv. ‘Junzao’ fruit extracts obtained by ultrasound-assisted extraction (UAE) alone (N), pectinase-assisted UAE (G), cellulase-assisted UAE (X), and composite enzyme-assisted UAE (GX). (**A**) TPC; (**B**) TFC. Values are expressed as mean ± standard deviation (SD) of triplicate measurements (*n* = 3). The asterisk indicates a statistically significant difference (** *p* < 0.01).

**Figure 2:**
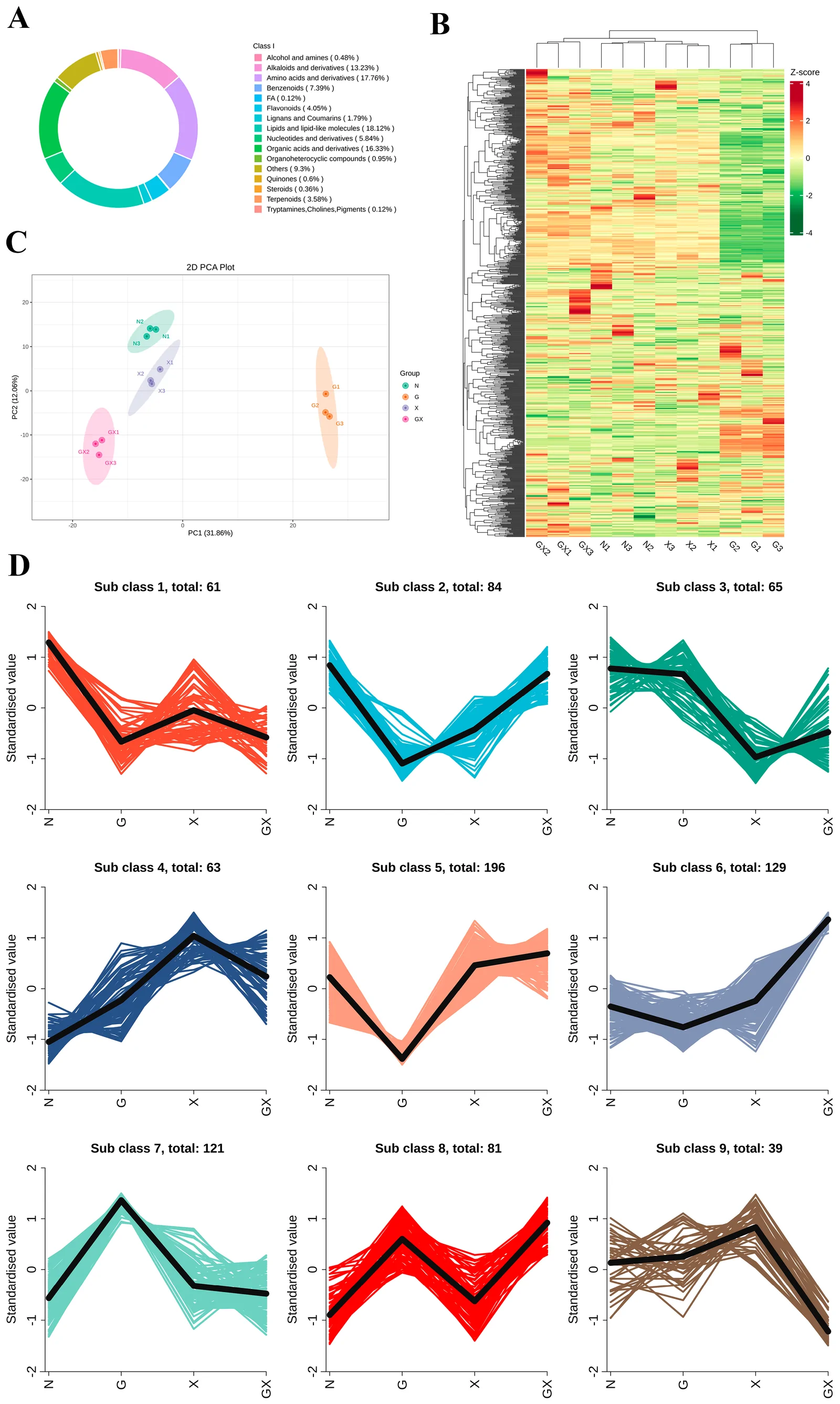
Analytical overview of metabolites detected in Junzao fruit extracts obtained by four extraction methods (N, G, X, and GX). (**A**) Circle chart showing the classification and proportion of metabolites identified across all samples. (**B**) Hierarchical clustering analysis (HCA) heatmap of metabolites across the four extraction groups. Rows represent individual metabolites, and columns represent samples. Red indicates relatively high abundance, whereas green indicates relatively low abundance. (**C**) Principal component analysis (PCA) score plot showing the distribution of samples. (**D**) K-means clustering analysis of all metabolites into nine clusters based on release patterns of metabolites.

**Figure 3:**
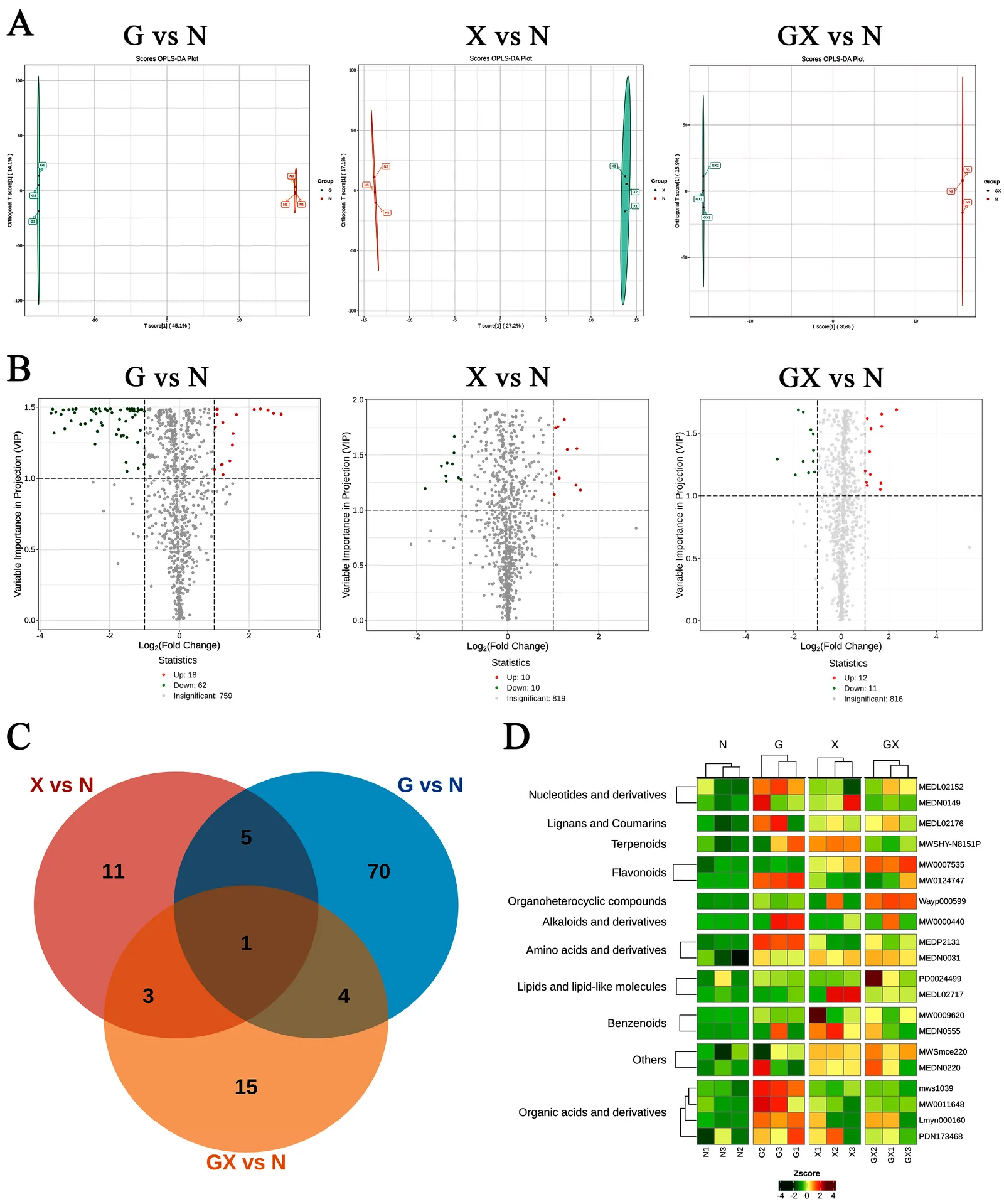
Orthogonal partial least squares discriminant analysis (OPLS-DA) of Junzao fruit extracts in different pairwise comparisons: G vs. N, X vs. N, and GX vs. N. (**A**) OPLS-DA score plots showing the separation for each pairwise comparisons. (**B**) Volcano plots showing differentially extracted metabolites (DEMs) for each pairwise comparison. Red dots indicate metabolites with significantly higher extraction efficiency, green dots indicate metabolites with significantly lower extraction efficiency, and gray dots indicate metabolites with no significant difference. (**C**) Venn diagram showing the number of shared and specific DEMs among the G vs. N, X vs. N, and GX vs. N comparisons. (**D**) Chemical classification and distribution of 20 higher yield DEMs across all enzyme-assisted methods (G, X, and GX) compared to ultrasound extraction alone (N). Red indicates relatively high abundance, whereas green indicates relatively low abundance.

**Figure 4:**
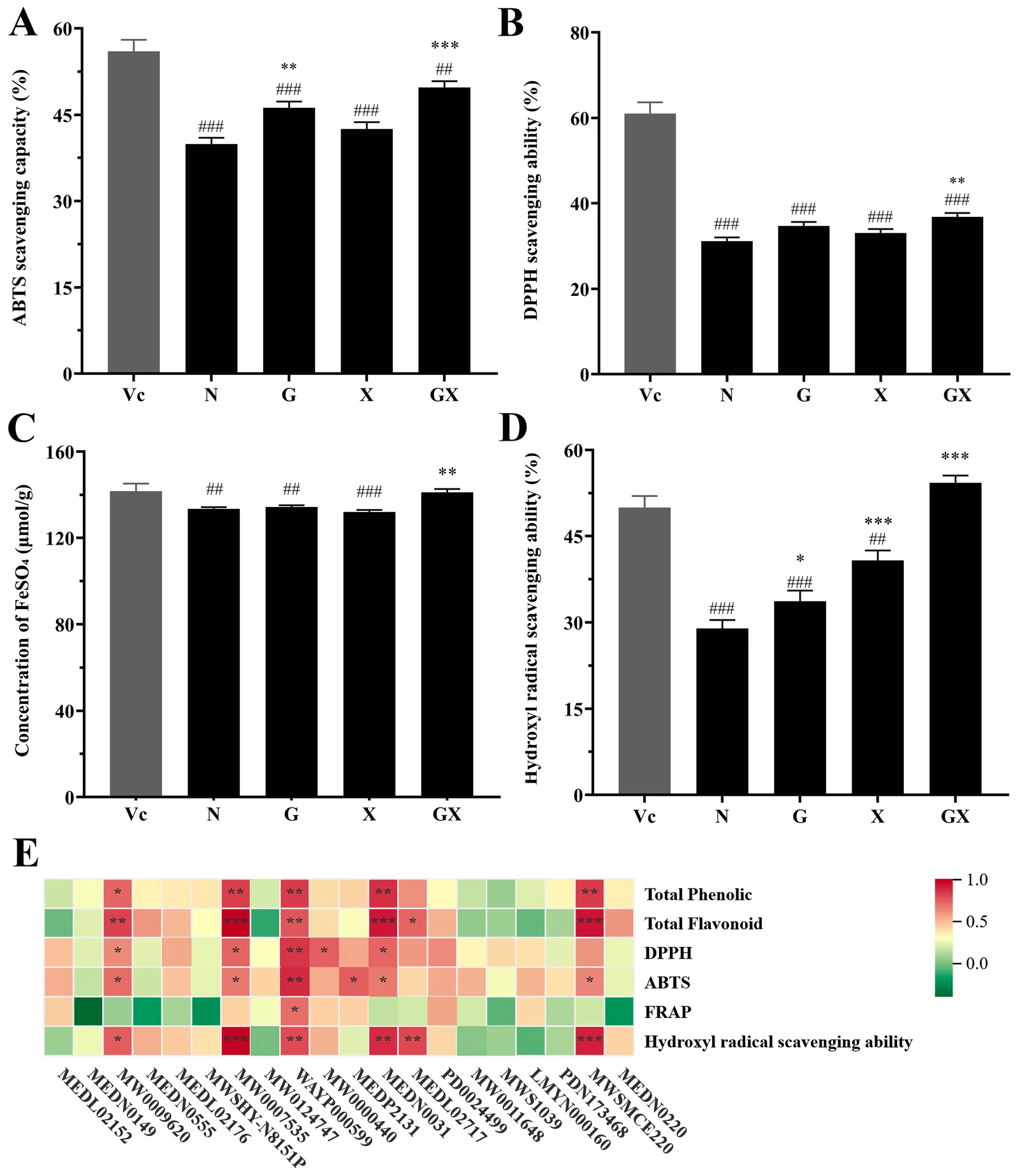
Antioxidant activities and correlation analysis of Junzao fruit extracts obtained by four extraction methods (N, G, X, and GX). (**A**) 2,2′-azino-bis(3-ethylbenzothiazoline-6-sulfonic acid) (ABTS) radical scavenging capacity. (**B**) 2,2-diphenyl-1-picrylhydrazyl (DPPH) radical scavenging capacity. (**C**) Ferric reducing antioxidant power (FRAP). (**D**) Hydroxyl radical scavenging capacity. Compared to the control group (N), asterisks denote statistical significance (* *p* < 0.05; ** *p* < 0.01; *** *p* < 0.0001). Compared to the positive controls (Vc), hash symbols denote statistical significance (^##^ *p* < 0.01; ^###^ *p* < 0.0001). (**E**) Spearman correlation heatmap between 20 higher yield DEMs and six bioactivity indicators (TPC, TFC, ABTS, DPPH, FRAP, and hydroxyl radical scavenging activity). Red and green colors indicate positive and negative correlations, respectively.

## Data Availability

The original contributions presented in this study are included in the article/Supplementary Materials. Further inquiries can be directed to the corresponding authors.

## Supplementary Materials

The following supporting information can be downloaded at https://www.mdpi.com/article/10.3390/antiox15101244/s1, Figure S1: Calibration curves for the determination of total phenolic content and total flavonoid content. (A) Total phenolic content was expressed as milligrams of gallic acid equivalents per gram of dry weight (mg GAE/g DW). (B) Total flavonoid content was expressed as milligrams of rutin equivalents per gram of dry weight (mg RE/g DW); Figure S2: Overlaid total ion chromatograms (TICs) of QC and sample extracts in negative (A) and positive (B) ionization modes, demonstrating analytical reproducibility and system stability throughout the run; Figure S3: TICs of extracts obtained by four different extraction methods in negative and positive ion modes. (A) ultrasound-assisted extraction (UAE) (N), (B) pectinase-assisted UAE (G), (C) cellulase-assisted UAE (X), and (D) composite enzyme-assisted UAE (GX), respectively. The left and right columns correspond to the negative ion mode and positive ion mode, respectively; Figure S4: (A) Orthogonal partial least squares discriminant analysis (OPLS-DA) score plots showing separation among the four extraction groups. (B–D) Permutation tests for the OPLS-DA models in the pairwise comparisons: G vs. N; X vs. N, and GX vs. N; Figure S5: Box plots showing the raw signal intensities of 20 higher yield differentially extracted metabolites (DEMs) across all enzyme-assisted methods (G, X, and GX) compared to N. Table S1: All metabolites identified in *Ziziphus jujuba* cv. ‘Junzao’ fruit extracts obtained using different extraction methods; Table S2: Results of K-mean analysis; Table S3: All differentially extracted metabolites (DEMs) identified across three pairwise comparisons (G vs. N, X vs. N, and GX vs. N); Table S4: DEMs with significantly higher extraction efficiency in all three enzyme-assisted groups (G, X, and GX) compared with N group; Table S5: Spearman correlation analysis between antioxidant capacity and metabolites.
