# Supplementary material for: Enzyme-Assisted Ultrasound Extraction of Bioactive Compounds from Ziziphus jujuba Mill. cv. ‘Junzao’ Fruits: Untargeted Metabolomic Profiling Reveals Enhanced Antioxidant Capacity
Source: Antioxidants (Basel). 2026 Sep 26;15(10):1244. doi: 10.3390/antiox15101244 (PMC13700608; doi:10.3390/antiox15101244)
Supplement: Supplementary file 1 [file antioxidants-15-01244-s001.zip › Supplementary Figure S1-S5.pdf]

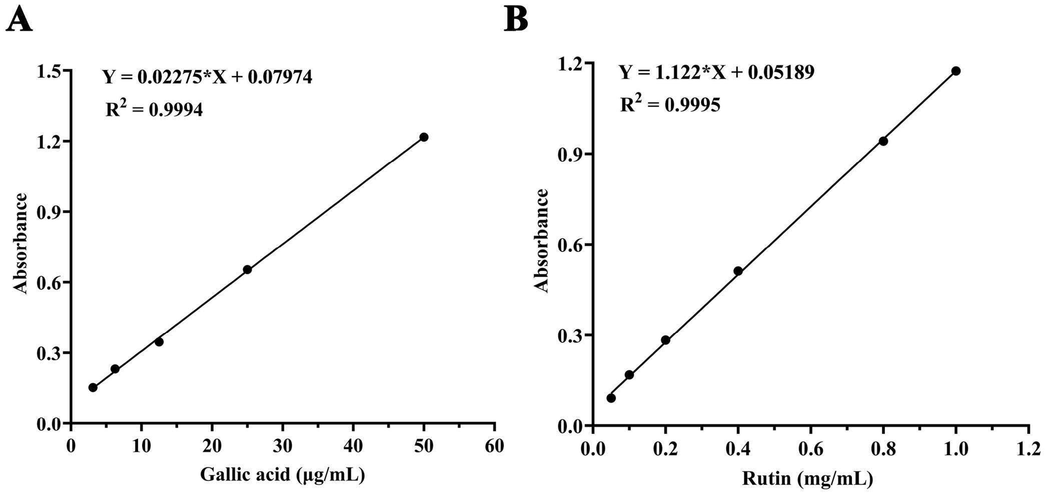

Figure S1: Calibration curves for the determination of total phenolic content and total flavonoid content. (A) Total phenolic content was expressed as milligrams of gallic acid equivalents per gram of dry weight (mg GAE/g DW). (B) Total flavonoid content was expressed as milligrams of rutin equivalents per gram of dry weight (mg RE/g DW).

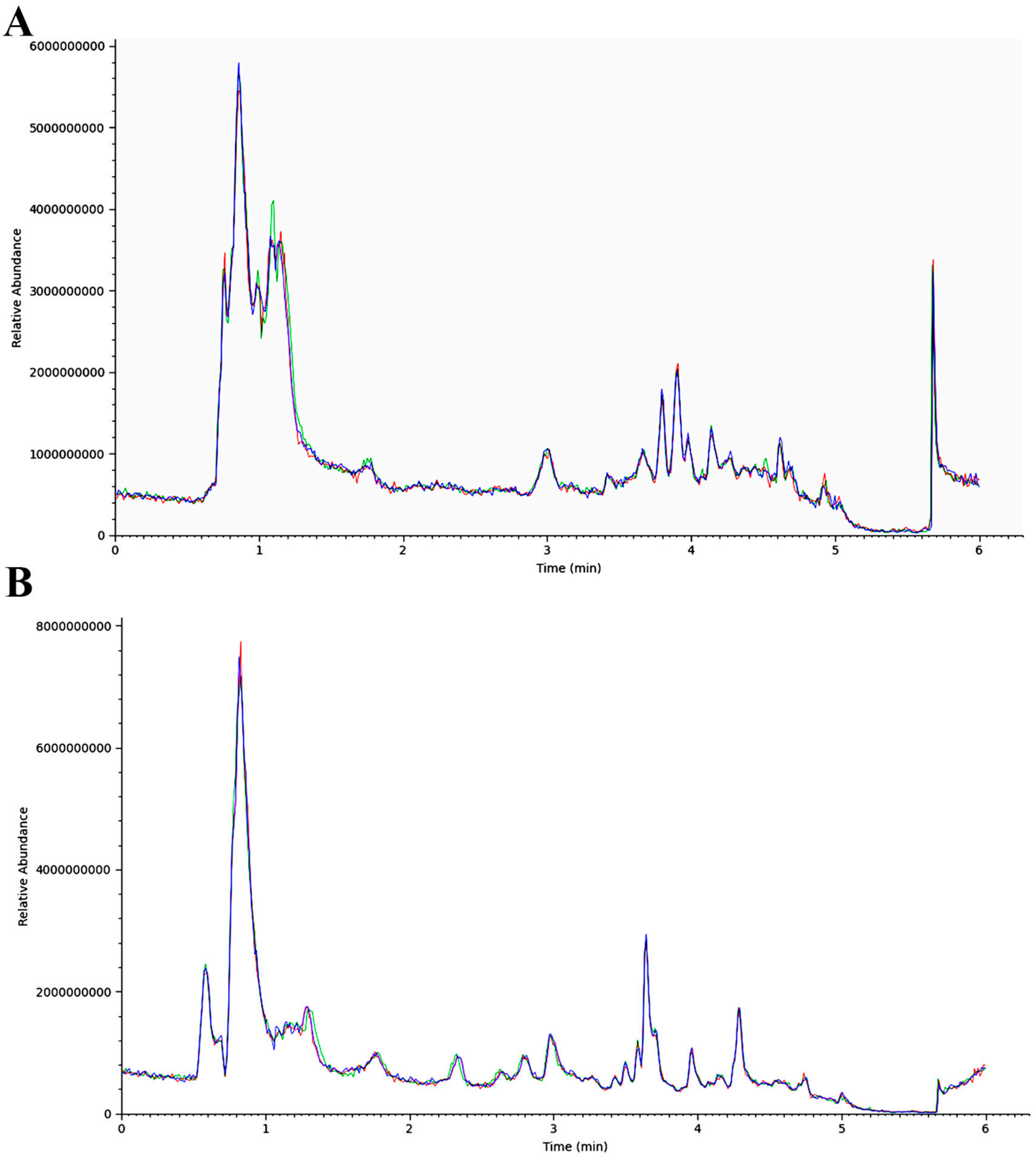

Figure S2: Overlaid total ion chromatograms (TICs) of QC and sample extracts in negative (A) and positive (B) ionization modes, demonstrating analytical reproducibility and system stability throughout the run.

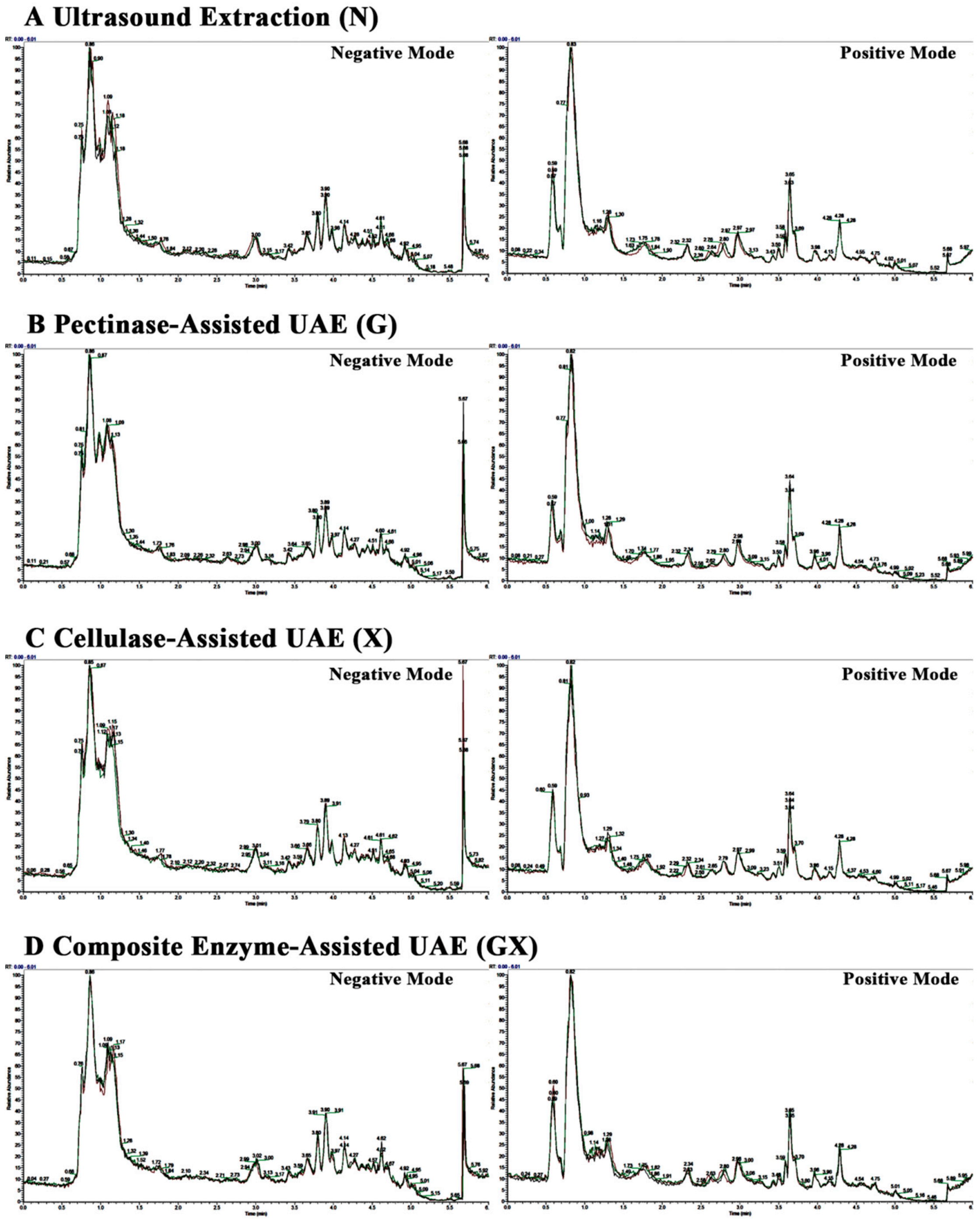

Figure S3: TICs of extracts obtained by four different extraction methods in negative and positive ion modes. (A) ultrasound-assisted extraction (UAE) (N), (B) pectinase-assisted UAE (G), (C) cellulase-assisted UAE (X), and (D) composite enzyme-assisted UAE (GX), respectively. The left and right columns correspond to the negative ion mode and positive ion mode, respectively.

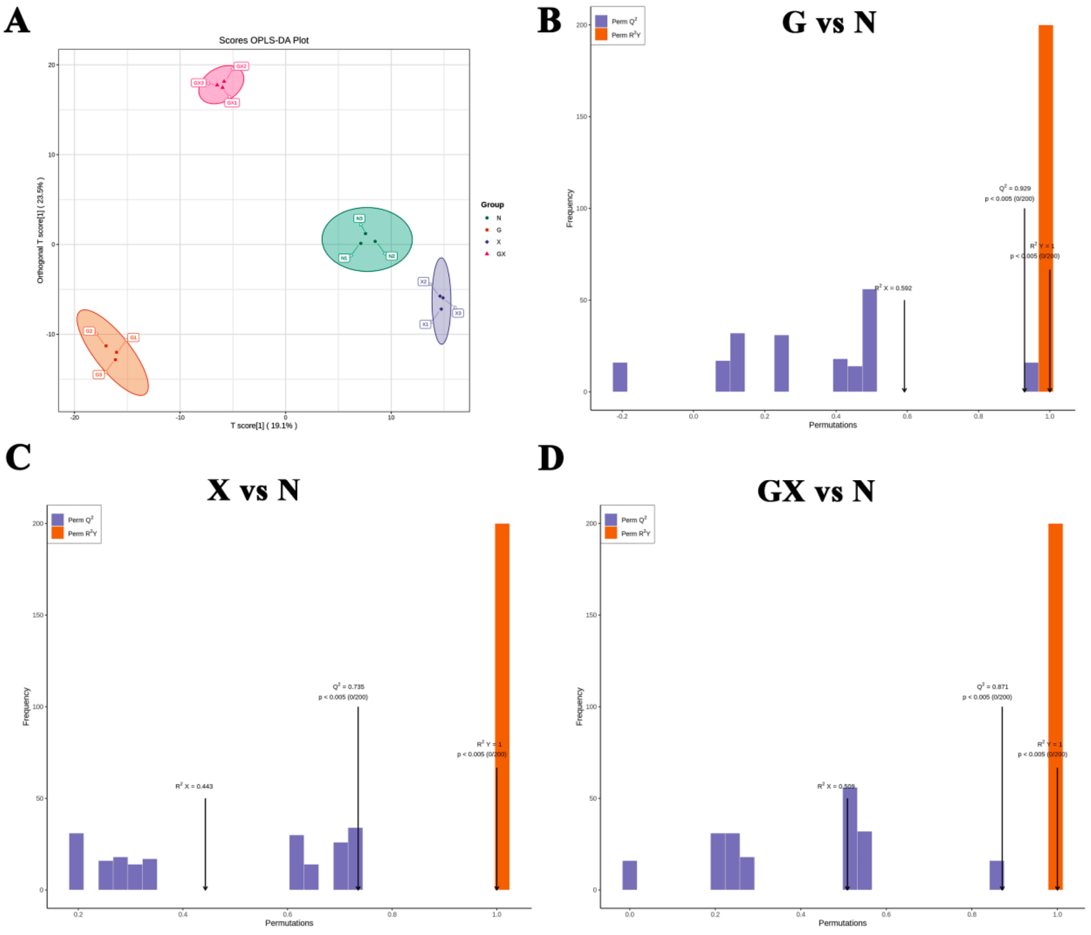

Figure S4: (A) Orthogonal partial least squares discriminant analysis (OPLS-DA) score plots showing separation among the four extraction groups. (B–D) Permutation tests for the OPLS-DA models in the pairwise comparisons: G vs. N; X vs. N, and GX vs. N.

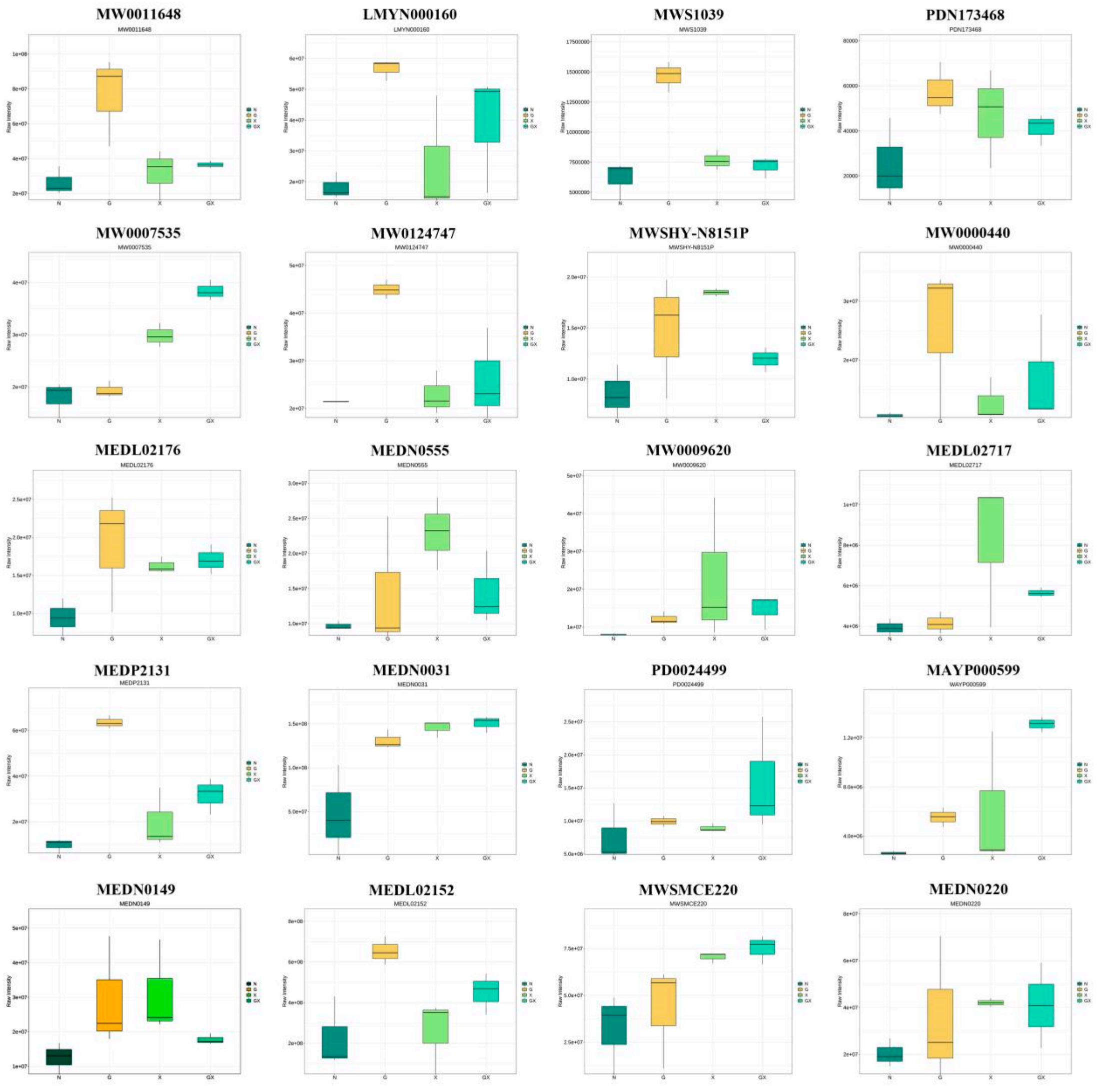

Figure S5: Box plots showing the raw signal intensities of 20 higher yield differentially extracted metabolites (DEMs) across all enzyme-assisted methods (G, X, and GX) compared to N.
